# Factors associated with online dating among college students engaging in casual sexual behavior in China: cross-sectional study

**DOI:** 10.3389/fpubh.2024.1417531

**Published:** 2024-08-23

**Authors:** Weiyong Chen, Zhongrong Yang, Qiaoqin Ma, Xin Zhou

**Affiliations:** ^1^Department of HIV/STD Control and Prevention, Zhejiang Provincial Center for Disease Control and Prevention, Hangzhou, Zhejiang, China; ^2^Huzhou Center for Disease Control and Prevention, Huzhou, Zhejiang, China

**Keywords:** HIV, online dating, cross-sectional study, aids, casual sexual behavior, factors, student, China

## Abstract

**Objective:**

This study aimed to investigate the factors associated with online dating among college students engaging in casual sexual behavior, by understanding these factors, targeted intervention measures can be formulated for relevant departments to help college students better manage their sexual health and offer useful reference for the development of sexual health education.

**Methods:**

A cross-sectional survey was conducted using a stratified cluster sampling method. Demographic and behavioral information was gathered through questionnaires for univariable and multivariable logistic regression analysis.

**Results:**

A total of 595 college students engaging in casual sexual behavior were included in the study, of whom 345 (57.98%) had found casual sexual partners through the internet. Multiple regression analysis indicated that male participants, those aged 20–21 years, those who had recently attended AIDS-themed lectures or health education classes at school, participants who were willing to engage in commercial sexual activities during online dating, participants who accepted sexual activities among men who have sex with men (MSM), those who reported having sexual intercourse with regular partners in the past year, participants who wanted to know if their online dating partners were HIV-diagnosed, those who had engaged in commercial sexual behavior in the past year and those who perceived themselves to be at risk of HIV infection were more likely to engage in online dating. Participants with general/disharmonious family relationships, those who consistently used condoms during casual sexual behavior and those who occasionally used condoms were less likely to engage in online dating.

**Conclusion:**

There were a certain extent proportion of casual partners among college students were sourced from the internet, indicating the profound influence of online dating on casual sexual behavior. Therefore, future research and intervention measures should focus on sexual health education and promotion on online dating platforms, strengthen regulations and guidance on college students’ online dating behavior, and raise awareness of HIV prevention in this group.

## 1 Introduction

Acquired immunodeficiency syndrome (AIDS) epidemic remains a major global public health issue; currently, AIDS cannot be cured and requires lifelong antiretroviral treatment (1–3). In recent years, AIDS among college students has attracted widespread global attention (4–6). Casual sexual behavior is frequent in college students and is closely related to the risk of sexually transmitted diseases (STDs) (7, 8). Therefore, it is especially important to investigate the variables associated with casual sexual behavior and human immunodeficiency virus (HIV) infection in college students. With rapid economic development and availability of the internet, the prolific use of the internet is having an increasingly significant impact on college students (9). Online dating has become one of the popular socializing methods among college students, and many students use various social media platforms and dating apps for online interaction and getting to know others (10, 11). The internet has expanded the horizons of sexual exploration for college students, encompassing activities like scheduling sexual encounters online, a trend that is becoming more widespread on a global scale (12). However, studies have found that online dating may lead to unsafe sexual behaviors, especially in cases with unknown sexual partners (11). Among college students, with the popularity and rapid development of the internet, online dating has become a popular way of socializing, contrasting significantly with traditional face-to-face dating. There are many differences between online and offline dating, which not only affect the social behavior and psychological state of college students as individuals but also have an impact on sexual health and HIV transmission risk. During online communication, personal identities and privacy can easily be concealed, leading to a lack of authenticity and transparency in the interaction process. Compared to traditional face-to-face interaction, the anonymity and convenience of online dating increase the likelihood of casual sexual behavior among college students, thereby increasing the risk of HIV transmission (12, 13). Through various social media platforms and dating apps, college students could easily meet people from different regions and backgrounds, providing them with convenient opportunities to expand their social circle and find suitable partners. This mode of interaction in a virtual environment would encourage college students to act more impulsively, increasing the risk of unsafe behaviors.

Understanding and identifying the factors that contribute to casual sexual behavior in online dating among college students can help develop more targeted sexual health education and HIV prevention measures to promote the healthy development of the college student population. College students face various psychological pressures and challenges, such as academic stress, interpersonal relationships, etc., which may lead some students to seek emotional support or psychological fulfillment through online dating. Additionally, college students often find themselves in different social circles, and the various social environments and interaction styles may influence their social behavior and dating habits. On virtual social networking platforms, individuals may be more inclined to let their guard down or portray a different side of themselves than in real life, and this type of communication interaction in this environment may make college students more prone to engaging in casual sexual behavior.

What are the factors associated with online dating among college students engaging in casual sexual behavior, and how do these factors contribute to the risk of HIV transmission in this population? It is expected that certain variables associated with online dating practices will be linked to an increased risk of engaging in unsafe sexual behaviors among college students. This study aimed to explore the factors associated with Chinese college students engaging in online dating and casual sexual behavior, and how these factors impact the risk of HIV transmission. Through conducting a cross-sectional study and deeply analyzing the relevant behavioral characteristics of college students in online dating, the study aims to more accurately identify the influencing factors related to casual behavior in order to provide specific recommendations and intervention measures for sexual health education and prevention efforts.

## 2 Materials and methods

### 2.1 Ethics approval

Consent to participate was obtained from each participant before data collection in this study. The research data for this survey are anonymous or de-identified, no identification of individual participants in any images of the manuscript or supplementary material is possible. The study protocol and consent procedure were approved by the Ethics Committee of the Zhejiang Provincial Center for Disease Control and Prevention (protocol number 2018–036).

### 2.2 Research design

We performed a cross-sectional survey among college students from 13 universities across 11 districts in Zhejiang Province from October to December 2018, including three universities in Hangzhou and one university in each of the other 10 districts (14). The selection of universities was recommended by the local Center for Disease Control and Prevention, and approved by the university, and a questionnaire survey is conducted with the cooperation of the university. In 2018, there were a total of 109 universities in Zhejiang Province, with 1.02 million students enrolled (excluding postgraduates). The study employed the random number table method to select three departments from each university, ensuring each department had a minimum of 800 students. Subsequently, the departments were stratified by grade level: with each grade level in a four-year program consisting of no fewer than 200 students, and each grade level in a three-year program comprising at least 267 students. Classes were then chosen within each grade level using the random number table method for the purpose of conducting surveys on all students in the selected classes.

### 2.3 Participants

**Figure 1:**
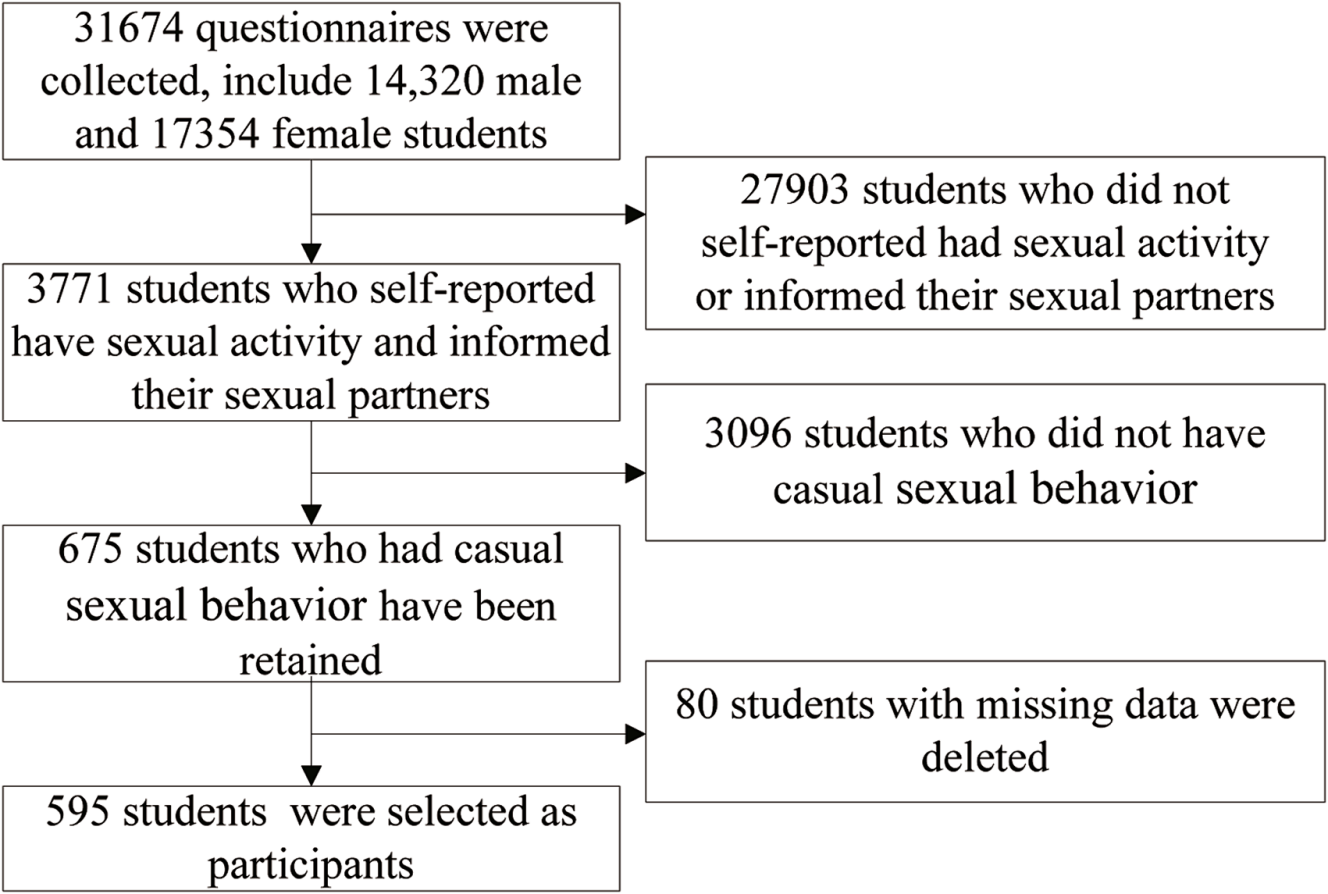
The flowchart for the inclusion and exclusion process.

On-campus students were directed by their teachers to complete an online electronic survey by scanning a QR code on their mobile phones. Off-campus students were instructed to complete the survey independently by following the links and instructions provided at the beginning of the questionnaire. The sample size calculation for this study was based on a cross-sectional survey sampling method. The prevalence of sexual behavior among college students was estimated to be around 15% (*p* = 0.15). By applying the formula *n* = 400*Q/P, where *Q* = 1-P, the necessary sample size for the study was determined to be 2,267 participants. In total, 31,674 students participated in the survey. Among the college students surveyed, there were 14,320 men and 17,354 women. A total of 3, 771 participants self-reported engaging in sexual activity in the past year and disclosed the source of their sexual partners. Among them, 675 self-reported engaging in casual sexual behaviors, accounting for 2.13% of the total number of students and 17.90% of the sexually active students. The inclusion criteria for the participants were college students who engaged in casual behaviors and disclosed the source of their casual partners, while the exclusion criteria included college students who engaged in casual behaviors but did not disclose the source of their casual partners, or those with missing variables. Finally, 595 participants were included in the study (Figure 1).

Participants were divided into two groups: online and offline. The online group (online dating group) included students with casual partners obtained through social media, online games, live streaming platforms, and other Internet-based methods. The offline group comprised students with casual partners obtained through entertainment venues, familiar acquaintances, or other non-internet-based channels.

### 2.4 Questionnaire content and quality control

The survey questionnaire was developed based on a review of the related literature (15, 16), discussions within the research team. We conducted a pilot survey of 30 students in a class at a university, and after the pre survey, we made adjustments to issues such as sexual behavior and HIV testing. We also conducted a consistency test on the self-efficacy in condom use. The survey questionnaire included general demographic characteristics, attitudes towards sexuality, sexual behavioral characteristics, HIV testing status, HIV risk perception, online dating, confidence in condom use, and other related topics. The survey was conducted anonymously using a standardized questionnaire, and the surveyors comprised professional staff from the local disease control and prevention centers and counselors from the surveyed university classes. They received uniform training before the survey. Prior to the survey, the surveyors explained the purpose, significance, methods, and privacy protection policy to the students. This information was included in the introduction of the survey questionnaire. Participants were informed that the survey was anonymous, aimed at formulating HIV and STDs prevention strategies for students, and that only group data would be analyzed.

Referring to the study by Hanna (16), a scale for measuring the self-efficacy in condom use was developed, which consisted of three main questions: whether the participant is confident in discussing condom use with a sexual partner before engaging in sexual activity, whether they feel confident in refusing sex or not engaging in sexual activity if the partner does not have a condom, and whether they feel confident in preparing a condom before engaging in sexual activity. Each question had five response options: “very confident,” “quite confident,” “confident,” “not confident,” and “not at all confident,” with corresponding scores of 3, 2, 1, 0, and-1, respectively. The participants were divided according to the total score into three groups: 9 points, 5–8 points, and < 4, a higher score indicates greater self-efficacy. The Cronbach’s alpha coefficient for this measurement was 0.812.

### 2.5 Definitions of the relevant indicators

Casual sexual behavior was defined as sexual activity with other men or women in the past year, excluding regular boyfriends or girlfriends. Commercial sexual behavior refers to sexual activities involving monetary transactions. Based on the sources of casual sexual partners in the past year, participants were divided into school students and non-students. Monthly living expenses were defined as the amount of money spent by the participants in each month. Sexual activities encompassed anal sex, vaginal sex, and oral sex. Condom usage during sexual activity was categorized into always used, occasionally used, and never used condoms.

### 2.6 Data analysis

SPSS software (version 21.0; SPSS Inc., Chicago, IL, United States) was used for data analysis. Variables such as age, sex, household registration, hometown, monthly living expenses, family relationships, attitudes towards sexuality, sexual behavior characteristics, HIV testing status, and HIV risk perception were presented as ratios or rates. The demographic characteristics of the college students who engaged in casual sexual behaviors and disclosed the source of their casual sexual partners were analyzed using the χ^2^ test. The independent variables included demographic characteristics, attitudes towards sexuality, HIV testing status, sexual behavioral characteristics, HIV risk perception, and self-efficacy in condom use. Factors associated with the participants’ engagement in online dating were analyzed using univariable logistic regression. Subsequently, variables with *p* < 0.2 were included in multivariable logistic regression analysis with the source of casual partners (whether engaging in online dating) as the dependent variable, and the “Enter” type of method was used in the multivariable regression. Control measures for confounding factors in this study primarily involve emphasizing random sampling when selecting participants, conducting a preliminary investigation prior to refining questionnaire design, implementing consistency checks on specific questions (e.g., assessing the effectiveness of condom use), and utilizing a multiple logistic regression model to address potential confounding factors through both univariate and multivariate analyses. Differences were considered statistically significant at *p* < 0.05.

## 3 Results

### 3.1 Demographic characteristics of the participants

A total of 595 college students engaging in casual sexual behavior were included in the study, of whom 345 (57.98%) had found casual sexual partners through the internet, the age of the participants was between 18 and 30 years old, with an average age of 20.15 ± 1.59 years; 250 (42.02%) engaged in non-internet-based casual sexual relationships, with an average age of 20.02 ± 1.43 years. There were statistically significant differences in sex and family relationships between the two groups (*p* < 0.05) (Table 1).

**Table 1:** Demographic characteristics with online dating among college students engaging in casual sexual behavior.

Variables	Offline dating group (*n* = 250, %)	Online dating group(*n* = 345, %)	*x^2^*	*P*
Age (yrs)				
≤19	95(38.0)	106(30.7)	5.954	0.051
20–21	116(46.4)	195(56.5)		
≥22	39(15.6)	44(12.8)		
Sex			7.055	0.008
Female	45(18.0)	36(10.4)		
Male	205(82.0)	309(89.6)		
Province of household registration			0.609	0.435
Zhejiang province	186(74.4)	246(71.5)		
Non-Zhejiang Province	64(25.6)	98(28.5)		
Hometown of origin			0.158	0.691
Rural area	114(45.6)	163(47.2)		
Town/City	136(54.4)	182(52.8)		
Monthly living expenses (CNY^*^)			3.135	0.209
≦1,000	64(25.6)	111(32.1)		
1,001–1,500	78(31.2)	102(29.6)		
≧1,501	108(43.2)	132(38.3)		
Family relationship			7.123	0.008
Harmonious	178(71.2)	278(80.6)		
General/disharmonious	72(28.8)	67(19.4)		

^*^CNY, Chinese Yuan.

### 3.2 Univariable analysis of factors associated with online dating among the participants

According to the results of the univariable analysis (Table 2), the variables with statistically significant differences included receiving AIDS-themed lectures or health education classes organized by the school in the past year (odds ratio, [OR]:1.57), undergoing AIDS risk self-assessment organized by the school in the past year (OR:1.63), being receptive to commercial sexual behavior (OR:2.65), being receptive to sexual activities among MSM (OR:3.06), having indulged in sexual activity with a regular partner in the past year (OR:1.85), expressing a desire to know whether the partner is diagnosed with HIV (OR:1.82), engaging in commercial sexual behavior in the past year (OR:4.73), self-perception of being at risk of HIV infection (OR:4.89), having a self-efficacy score of 9 for condom use (OR:1.65), and consistently using condoms (OR:0.16) or occasionally using condoms (OR:0.23)during casual sexual encounters.

**Table 2:** Univariable and multivariable analysis of online dating among college students engaging in casual sexual behavior.

Variables	Offline dating group	Online dating group	Univariable analysis		Multivariable analysis	
	*n* (%)	*n* (%)	OR (95%CI)	*p*	a*OR* (95%*CI*)	*p*
Age (yrs)						
≤19	95(38.0)	106(30.7)	1		Ref.	
20–21	116(46.4)	195(56.5)	1.51(1.05–2.16)	0.026	1.57(1.03–2.41)	0.038
≥22	39(15.6)	44(12.8)	1.01(0.61–1.69)	0.966	1.11(0.60–2.07)	0.734
Sex						
Female	45(18.0)	36(10.4)	1		Ref.	
Male	205(82.0)	309(89.6)	1.88(1.18–3.02)	0.009	3.20(1.65–6.20)	0.001
Family relationship						
Harmonious	178(71.2)	278(80.6)	1		Ref.	
General/disharmonious	72(28.8)	67(19.4)	0.60(0.41–0.87)	0.008	0.63(0.40–0.99)	0.043
Have you received AIDS-themed lectures or health education courses from the school in the past year						
No	91(36.4)	92(26.7)	Ref.		Ref.	
Yes	159(63.6)	253(73.3)	1.57(1.11–2.24)	0.011	1.68(1.01–2.81)	0.047
Have you learned about HIV/AIDS through the school network in the past year?						
No	71(28.4)	85(24.6)	Ref.		–	–
Yes	179(71.6)	260(75.4)	1.21(0.84–1.75)	0.303	–	–
Have you received any publicity about HIV testing from the school in the last year?						
No	90(36.0)	100(29.0)	Ref.		Ref.	
Yes	160(64.0)	245(71.0)	1.38(0.97–1.95)	0.07	0.83(0.50–1.38)	0.466
Have you received an HIV risk self-assessment conducted by the school in the past year?						
No	144(57.6)	157(45.5)	Ref.		Ref.	
Yes	106(42.4)	188(54.5)	1.63(1.17–2.26)	0.004	0.90(0.58–1.40)	0.627
Do you accept commercial sex?						
Do not accept/do not know	148(59.2)	122(35.4)	Ref.		Ref.	
Yes	102(40.8)	223(64.6)	2.65(1.90–3.71)	<0.001	1.60(1.06–2.41)	0.024
Do you accept sexual activities among men who have sex with men (MSM)?						
Do not accept/do not know	210(84.0)	218(63.2)	Ref.		Ref.	
Yes	40(16.0)	127(36.8)	3.06(2.05–4.56)	<0.001	3.47(2.00–6.02)	<0.001
Casual partner types						
School student	171(68.4)	211(61.2)	Ref.		Ref.	
Non-student	79(31.6)	134(38.8)	1.38(0.98–1.94)	0.069	1.51(1.00–2.28)	0.051
Have you had sex with a stable partner in the past year						
No	97(38.8)	88(25.5)	Ref.		Ref.	
Yes	153(61.2)	257(74.5)	1.85(1.30–2.63)	<0.001	1.66(1.09–2.52)	0.018
Condom use with casual partners						
Never used	21(8.4)	110(31.9)	Ref.		Ref.	
Occasional used	110(44.0)	134(38.8)	0.23(0.14–0.40)	<0.001	0.43(0.23–0.79)	0.007
Every time used	119(47.6)	101(29.3)	0.16(0.10–0.28)	<0.001	0.27(0.14–0.50)	<0.001
Whether discussed using condoms during sexual intercourse?						
No	72(28.9)	97(28.2)	Ref.		–	–
Yes	177(71.1)	247(71.8)	1.04(0.72–1.49)	0.848	–	–
Do you want to know that your sex partner may be diagnosed with HIV?						
No	134(53.6)	134(38.8)	Ref.		Ref.	
Yes	116(46.4)	211(61.2)	1.82(1.31–2.53)	<0.001	1.59(1.08–2.34)	0.02
Whether had engaged in commercial sex in the past year						
No	216(86.4)	195(56.5)	Ref.		Ref.	
Yes	34(13.6)	150(43.5)	4.73(3.16–7.10)	<0.001	2.29(1.40–3.76)	0.001
Do you think that you are at risk of contracting HIV?						
No/Do not know	234(93.6)	256(74.2)	Ref.		Ref.	
Yes	16(6.4)	89(25.8)	4.89(3.21–7.44)	<0.001	2.39(1.25–4.57)	0.008
Measurement of self-efficacy in condom use (scores)						
Less than or equal to 4	71(28.4)	78(22.6)	Ref.		Ref.	
5–8	86(34.4)	98(28.4)	1.04(0.67–1.60)	0.868	1.10(0.65–1.84)	0.731
9	93(37.2)	169(49.0)	1.65(1.10–2.49)	0.016	1.16(0.70–1.93)	0.562
Have you received voluntary counseling and testing in the past year?						
No	236(94.4)	294(85.2)	Ref.		Ref.	
Yes	14(5.6)	51(14.8)	2.92(1.58–5.41)	0.001	1.55(0.75–3.21)	0.243
How about condom use during sex with a regular partner in the past year? (*n* = 410)						
Never used	17(111.1)	108(42.0)	Ref.		–	–
Occasional used	74(48.4)	83(32.3)	0.18(0.10–0.32)	<0.001	–	–
Every time used	62(40.5)	66(25.7)	0.17(0.09–0.31)	<0.001	–	–

Of the 595 college students engaging in casual sexual behavior, 410 (68.9%) had a regular sexual partner in the past year. Further analysis revealed that among college students with regular sexual partners in the past year, 31.2% (128/410) consistently used condoms. The analysis also showed statistically significant differences in using condoms every time/sometimes/often and never using condoms when engaging in sexual behavior with a regular partner (OR = 0.17, OR = 0.18, respectively).

### 3.3 Multivariable analysis of factors associated with online dating among the students

After adjusting variables with *p* < 0.2 from the univariable analysis into the multivariable logistic regression model, the results (Table 2) indicated that compared to female college students, the proportion of male college students engaging in online dating has increased by 220% (adjusted odds ratio [aOR]: 3.20; 95% confidence interval [CI]: 1.65–6.20); compared to the age group of 19 years and below, the proportion of participants aged 20–21 years engaging in online dating has increased by 57% (aOR:1.57;95% CI:1.03–2.41); participants who have received AIDS-themed lectures or health education classes offered by the school in the past year showed a 68% increase in online dating (aOR:1.68;95% CI:1.01–2.81); participants receptive to commercial sexual behavior demonstrated a 60% increase in online dating (aOR:1.60;95% CI:1.06–2.41); participants who accepted sexual activities among MSM exhibited a 247% increase in online dating (aOR:3.47;95% CI:2.00–6.02); participants who indulged in sexual activity with a regular partner in the past year showed a 66% increase in online dating (aOR:1.66;95% CI:1.09–2.52); participants desiring to know if their partner was diagnosed with HIV demonstrated a 59% increase in online dating (aOR:1.59;95% CI:1.08–2.34); participants who engaged in commercial sexual behavior in the past year showed a 129% increase in online dating (aOR:2.29;95% CI:1.40–3.76); and participants who perceived themselves at risk of HIV infection showed a 139% increase in online dating (aOR:2.39;95% CI:1.25–4.57). Compared to participants with harmonious family relationships, participants with general/disharmonious family relationships demonstrated a 37% decrease in online dating (aOR:0.63;95% CI:0.40–0.99); compared to participants who never used condoms during casual sexual encounters, participants who consistently used condoms or occasionally used condoms showed a decrease of 73% (aOR:0.27;95% CI:0.14–0.50) and 57% (aOR:0.43;95% CI:0.23–0.79) in online dating, respectively.

## 4 Discussion

In recent years, the rapid spread of the internet has made life considerably convenient. This cross-sectional survey of college students from Zhejiang Province effectively reflects the characteristics of college students engaging in casual behaviors through online dating. The results of this study indicated that 57.98% of the 595 participants engaged in online dating, suggesting that with the widespread availability of the internet. This research can help public health institutions better understand the behavioral patterns and health needs of college student populations, thereby enabling targeted development of preventive measures and educational programs. At the same time, by exploring the relationship between online dating and casual sexual behavior, public health departments can better understand how these behaviors impact the spread of infectious diseases, particularly sexually transmitted infections. This will aid in implementing more targeted infectious disease control strategies, including raising students’ health awareness, promoting safe sexual behaviors, and increasing investment in healthcare resources. In conclusion, this study is of significant importance for improving health management and infectious disease control among college student populations, contributing to the enhancement of public health standards and ensuring the health and safety of college student populations.

The proportion of male college students engaging in online dating was higher than that of female college students (the proportion of male college students engaging in online dating increased by 220% compared to female college students). This reflects the changing attitudes and behavioral habits of male and female students towards online dating in the society. Traditionally, men are expected to actively seek social interactions and relationships, while women are expected to be more conservative and cautious. This could have led to faster growth in the use of online dating platforms among male college students. Additionally, the influence of the social environment and culture on the online dating behavior of men and women should be considered. Research suggests that certain social platforms may be preferred by male college students, or in some social circles, male college students may face more pressure or demands for social interaction (17, 18).

The present study also suggests an increase in online dating activities among those in the age group of 20–21 years. Compared with participants aged 19 years and below, the proportion of participants in the 20–21 years age group who engaged in online dating increased by 57%. This phenomenon may reflect the tendency of this age group to use online dating platforms for social networking and companionship. Students in this age group may have transitioned from school to work or independent living, leading to a reevaluation and exploration of their personal identities and social relationships. This drives them to actively utilize online dating platforms to meet their social needs, thus making them more likely to expand their social circles and seek new friends or partners through online platforms (9, 19). However, while actively seeking new social opportunities, it is important for college students to be aware of the potential risks of online dating and maintain rationality and vigilance.

Furthermore, participants who had received special lectures or health education classes on HIV/AIDS in school in the past year showed a 68% increase in online dating. This may reflect the positive impact of health education on social behavior. Health education courses may increase participants’ emphasis on their health behavior and self-protection awareness, making them more likely to pay attention to health conditions and risk factors when seeking friends or partners, and using online platforms to find people with similar health perspectives (20). However, while increasing sexual health awareness, it is necessary to ensure the accuracy and comprehensiveness of the educational content to provide effective guidance and assistance to individuals (19). Additionally, health education may encourage people to discuss health topics, including sexual health and risks of infectious diseases. Individuals who have received health education may be more willing to share related experiences and information online. Therefore, health education may play a role in social behavior and online dating, guiding people to pay more attention to health issues and engage in social interactions and friendships more responsibly, thus providing useful references and insights for the development of HIV/AIDS health education courses and social policies (21).

The anonymity and openness of the internet may provide a more convenient social platform for MSM community (22). The results of this study revealed a 247% increase in the proportion of participants who accepted sexual activities among MSM. This increase may reflect the gradual improvement in society’s tolerance and understanding of sexual orientation. Compared with traditional social methods, online dating platforms offer individuals with specific sexual orientations a wider range of choices and a more open social environment (18). Although societal awareness of MSM rights is gradually increasing, discrimination and misunderstanding towards MSM still persist in certain regions and social groups (23). Therefore, despite providing a more open social environment for MSM, efforts are still needed to eliminate discrimination and bias towards sexual orientation to create a more inclusive and understanding societal environment.

With the rising prevalence of social media and dating apps, an increasing number of people are choosing to seek new sexual partners online rather than confining themselves to fixed interpersonal relationships in real life. The results of this study indicate that the proportion of participants who reported indulging in sexual activities with fixed sexual partners in the past year while engaging in online dating increased by 66%. This trend reflects the influence of rapid social and technological developments on interpersonal relationships and sexual behaviors. These data indicate growing acceptance and trust in online dating platforms. The use of online dating platforms may increase the risk of STDs because while engaging in anonymous online dating, individuals may overlook concerns about sexual health and safety (21). Therefore, while enjoying the convenience of technology, it is important to pay attention to sexual health and real-life interactions to balance the relationships between the virtual and real world.

This study indicate that the proportion of participants who accepted commercial sexual activities and engaged in commercial sexual activities in the past year, while engaging in online dating increased by 60 and 129%, respectively. These data may reflect a certain degree of change in societal attitudes towards sex work and sexual health. Individuals engaged in commercial sexual activities may face a range of health issues and are susceptible to gender discrimination and stigmatization (24). Therefore, more attention and resources are required to safeguard their rights and health. These findings suggest that there has been a shift in societal attitudes towards individuals engaged in sex work and commercial sexual activities and that there is a need for increased attention and discussion to ensure that the rights and health of those engaged in sex work are adequately protected.

With increasing spread of the knowledge regarding prevention of HIV and other STDs, people have become more willing to learn about the health status of their potential partners, especially when it comes to sexual health-related issues. This may also indicate that individuals place greater emphasis on their own health and safety and are willing to actively seek information about the health status of potential partners. According to the results of this study, the proportion of participants who wanted to know if their potential partners were diagnosed with HIV, as well as the proportion of participants who perceived themselves to be at risk of HIV infection among students engaging in online dating, increased by 59 and 139%, respectively. This trend reflects the importance of transparency in online dating. People are more willing to establish relationships with those who are open about their health status, which may help them to build honest and healthy relationships. However, excessive emphasis on others’ health status may lead to discrimination and exclusion, and it is important to balance personal rights and privacy protection. Overreliance on self-reported health status may also pose a risk of HIV infection (25). Therefore, while the importance of sexual health and HIV prevention is widely recognized in the society, it is also necessary to pay attention to the balance of personal privacy and rights as well as to establish healthy patterns for dating and interpersonal relationships.

Individuals raised in a harmonious family environment may have stronger self-esteem and confidence, making it easier for them to establish positive and healthy interpersonal relationships without relying excessively on the internet to seek social satisfaction. Our results revealed that compared to participants with harmonious family relationships, the proportion of participants from general or disharmonious family relationships who engaged in online dating decreased by 37%. This result may reflect the profound impact of family relationships on social behavior. Research has shown that good family relationships help promote students’ self-control and reduce risky sexual behaviors (26, 27). Therefore, while attention is given to online dating, it is important to emphasize attention and support for family relationships to foster healthy and harmonious social skills in individuals.

Compared with individuals who never used condoms during casual sexual encounters, the proportion of participants who consistently or occasionally used condoms during these encounters decreased by 73 and 57%, respectively, among students who engaged in online dating. This result may reflect the relationship among concerns for sexual safety, health, and behavioral choices. Efforts to promote sexual health primarily involve health education and individual-level interventions based on peers or skills (28). As sexual education and health promotion activities become more prevalent, an increasing number of individuals develop a deeper understanding of sexual health issues and are more willing to take relevant protective measures (such as consistently using condoms during sexual activities, reducing the number of sexual partners, or having a permanent sexual partner) to reduce potential infection risks associated with sexual behavior (29, 30). However, a decrease in the proportion of individuals consistently using condoms may indicate a cognitive bias or underestimation of personal risks related to sexual health issues. This emphasizes the need to strengthen the promotion and education of sexual health to increase people’s focus on sexual safety. These results remind us that sexual safety and health are important aspects of social behavior. It is crucial to pay attention to and guide individuals’ awareness and behavioral choices regarding sexual health issues, as well as to enhance their self-protection awareness.

This study has several limitations. First, as this was a cross-sectional survey, data were collected from the participants from October to December 2018, the study only observed various factors and outcomes at a certain time point and did not involve follow-up of the study participants, and changes and developmental trends over time were not observed, which may affect the relevance of the findings due to changes in online dating behaviors and social dynamics. Additionally, the cross-sectional nature of the study limits the ability to establish causal relationships between the identified factors and online dating behaviors. Meanwhile, random sampling was performed in this study, which may not fully represent college students, leading to selection bias in the study. However, this study conducted univariate and multivariate logistic regression analyses to reduce the impact of confounding factors. Moreover, there may be recall bias since participants were asked to recall their behaviors over the past year, leading to limited generalization of the results. Therefore, our results require further validation in multicenter, prospective, randomized cohort studies.

## 5 Conclusion

In this study of 595 college students who reported engaging in casual sexual behavior, a certain extent proportion of their casual partners were sourced from the internet, indicating the profound influence of online dating on college students’ casual sexual behavior. Male college students and those aged 20–21 years were more likely to engage in casual sexual behaviors through online dating. Additionally, college students who attended AIDS-themed lectures, accepted commercial sex, or accepted sexual activities among MSM were more likely to engage in casual sexual behavior through online dating. It is crucial to emphasize the factors (such as methods to prevent HIV transmission, the usage of condoms being promoted, reduce sexual partners or maintain stable sexual partners, etc.) that must be considered to encourage sexual health in digital media, and strengthen sexual health education in families and schools for college students. Future research aimed at enhancing sexual health among college students should expand its scope to investigate the enduring impact of casual sexual behavior and online dating on sexual health. Moreover, in the realm of sexual health education through digital platforms, studies could delve into strategies for optimizing the dissemination of sexual health information via social media and online platforms to steer youth toward healthier sexual practices. Subsequent research endeavors should delve into crafting sexual health policies that are comprehensive and impactful, with a focus on supporting college students in upholding healthy sexual behaviors in the digital era.

## Data Availability

The raw data supporting the conclusions of this article will be made available by the authors, without undue reservation.
